# EMG Muscle Activation Pattern of Four Lower Extremity Muscles during Stair Climbing, Motor Imagery, and Robot-Assisted Stepping: A Cross-Sectional Study in Healthy Individuals

**DOI:** 10.1155/2019/9351689

**Published:** 2019-03-25

**Authors:** Damaris E. Geiger, Frank Behrendt, Corina Schuster-Amft

**Affiliations:** ^1^Institute of Physiotherapy, Zurich University of Applied Sciences, Winterthur, Switzerland; ^2^Research Department, Reha Rheinfelden, Rheinfelden, Switzerland; ^3^Institute for Rehabilitation and Performance Technology, Bern University of Applied Sciences, Burgdorf, Switzerland; ^4^Department of Sport, Exercise and Health, University of Basel, Basel, Switzerland

## Abstract

**Background:**

Stair climbing can be a challenging part of daily life and a limiting factor for social participation, in particular for patients after stroke. In order to promote motor relearning of stair climbing, different therapeutical measures can be applied such as motor imagery and robot-assisted stepping therapy. Both are common therapy measures and a positive influence on the rehabilitation process has been reported. However, there are contradictory results regarding the neuromuscular effect of motor imagery, and the effect of robot-assisted tilt table stepping on the EMG activation compared to stair climbing itself is not known. Thus, we investigated the EMG activity during (1) a stepping task on the robot-assisted tilt table Erigo, (2) motor imagery of stair climbing, and (3) real stair climbing in healthy individuals for a subsequent study on patients with lower limb motor impairment. The aim was to assess potential amplitude independent changes of the EMG activation as a function of the different conditions.

**Methods:**

EMG data of four muscles of the dominant leg were recorded in m. rectus femoris, m. biceps femoris, m. tibialis anterior, and m. gastrocnemius medialis. The cross-correlation analysis was performed to measure similarity/dissimilarity of the EMG curves.

**Results:**

The data of the study participants revealed high cross-correlation coefficients comparing the EMG activation modulation of stair climbing and robot-assisted tilt table stepping in three muscles except for the m. gastrocnemius medialis. As the EMG activation amplitude did not differ between motor imagery and the resting phase the according EMG data of the motor imagery condition were not subjected to a further analysis.

**Conclusion:**

Robot-assisted tilt table stepping, but rather not motor imagery, evokes a similar activation in certain leg muscles compared to real stair climbing.

## 1. Introduction

For a high proportion of poststroke patients, the largest restrictions for themselves and their families are due to the limitation of activities and participation [1]. Stair climbing often seems to be a limiting factor for outdoor activities [2]. It was found that 25 out of 40 poststroke patients had difficulties related to leaving the house [3]. For instance, only about 5% of nonwalking early ischemic stroke patients were able to climb stairs independently at discharge from a rehabilitation hospital [4]. Even six months poststroke patients named stair climbing as a difficulty in basic activity of daily living [5]. However, there is evidence for promising and potentially beneficial rehabilitation measures to improve aspects of gait [6] and hence of stair climbing [7].

Robot-assisted training is a relatively novel approach in the rehabilitation of upper limb or gait recovery [1] and has rapidly evolved for more than a decade. This kind of training makes use of robotic devices that support whole body or limb movements by applying certain desired motion sequences. For instance, the robotic use for gait rehabilitation is classified according to the method being applied. The so-called “end-effector robots” only move feet, whereas “exoskeletal robots” apply control throughout the gait cycle by moving hip, knee, and ankle joints [8]. Robot-assisted training enables a greater number of repetitive tasks to be practiced in a consistent and controllable manner. It provides the possibility of a repetitive, interactive, high-intensity, and task-specific treatment of a limb [6] and can potentially improve motor recovery [9] due to interhemispheric connectivity and brain reorganization processes which were demonstrated to be induced by such training [10]. Especially in gait rehabilitation, the advantage of a robotic device is the combination of repeated and body weight supported practice which can enhance early mobilization after brain injury [11, 12] and the fact that while foot motion is guided by the device the remaining degrees of freedom are not restricted [13]. Robot-assisted training was also found to be a safe and effective way to verticalise bedridden poststroke patients [14]. Muscle activation of healthy subjects during robot-assisted gait training has been found to produce comparable rhythmic and phasic patterns to normal floor walking, which showed the potential role in gait rehabilitation in patients with an impaired central nervous system [13].

Motor imagery (MI) is a well-known method and has received increased attention as a training approach in the rehabilitation of stroke patients [15–17]. It was defined as a cognitive process in which a subject imagines to perform a movement and during which the representation of a specific motor action is internally activated without any motor output [18]. Parallel patterns of brain activation are systematically reported during the physical performance and MI of the same action [18]. In stroke rehabilitation, MI is commonly used for the rehabilitation of upper limb functions and has furthermore shown its effectiveness in the improvement of ambulation and activities of daily living functions [19, 20]. Malouin et al. found that the vividness of motor imagery in stroke patients is even similar to that of age-matched healthy persons [21]. It is typically combined with traditional task-specific training [6]. Furthermore, this training method can be performed by the patient alone after some familiarization and instruction [22]. Because the process of imagery is not dependent on the ability to perform a movement, MI can be implemented early in rehabilitation in order to train motor preparation and thus facilitate physical recovery [23]. Although MI is an established method for the recovery of selected motor functions and the effect on neuroplasticity in related brain areas is well-known [10, 24] the general effect on the peripheral motor system due to MI is not yet fully understood. Electromyography (EMG) studies on the muscular activity during MI tasks revealed varying results [25–28]. Some studies reported a subliminal EMG muscle activity during MI [27, 28]. For instance, during imaged and real tiptoe rises, a similar EMG muscle activity pattern of m. gastrocnemius medialis and m. rectus femoris could be found in healthy participants and stroke patients in at least one of the four investigated muscles [29]. In healthy sports students, a significant higher muscular activation was found during MI of elbow flection compared to the rest condition [30]. But further evidence is in disagreement with these findings as other studies did not report muscular activation during MI tasks, e.g., squatting exercises in healthy adults [31]. However, MI was included in this study as it was recently found that MI may have a beneficial task-specific effect on gait function in subacute stroke patients [23]. Applied research on MI practice in rehabilitation has already evaluated its efficacy in improving aspects of motor performance [32–34].

The aim of the present study was to assess the degree of similarity in the EMG profiles comparing three different conditions: (1) a stepping task on the robot-assisted tilt table Erigo, (2) a MI task of stair climbing, and (3) real stair climbing in four lower extremity muscles in healthy individuals to also define the normal, unimpaired activation in these three conditions. The results could subsequently serve as a basis for further investigations with impaired patients in order to enhance both therapeutical measures and to allow for a more targeted use.

We hypothesized that a similar EMG activation would occur during stair climbing and robot-assisted tilt table stepping. In contrast, based on the available literature it was unclear whether to expect a similar EMG activation as a result of motor imagery of such a complex movement as stair climbing.

## 2. Materials and Methods

Twelve healthy adult volunteers (8 females, 4 males; mean age 50.3 ± 14.6 years, age range 30-73 years) participated in the study. All participants were recruited among the clinic staff. None of the recruited volunteers was excluded or was experienced in using the Erigo. The right body side was the dominant side in eleven volunteers tested with the Edinburgh Handedness Inventory [35]. Volunteers could not be included if they had or suffered from recent injuries of upper or lower limb (<1 year), impairment in balance abilities, impairment of gait ability (e.g., walking aid), impairment in cardiopulmonary function, skin irritations, or acute pain. All procedures were approved by the local ethics committee (Ethics Committee Northwest and Central Switzerland, reference number 2016-00837). The study was conducted in accordance with the Declaration of Helsinki and Good Clinical Practice guidelines and written informed consent was obtained from all volunteers prior to data collection start. Sample size determination was based on two studies on the muscular activity during robot-assisted walking [13] and during MI [29]. Both included nine healthy subjects each. With regard to potential drop-outs, we decided to record the data of twelve participants.

The muscle activation was measured using disposable, self-adhesive dual electrodes (Ag/AgCL) (Noraxon, USA). Prior to placing the electrodes, skin preparation was performed to reduce input impedance of the recording site and consisted of shaving and cleansing with alcohol [36]. Once the alcohol had dried, the electrodes were placed on four gait relevant muscles of the lower extremity on the dominant leg [13, 37, 38]: m. rectus femoris, m. biceps femoris (BF), m. tibialis anterior, and m. gastrocnemius medialis, considering the SENIAM recommendations for skin preparation and electrode placement [39]. EMG data were recorded using a wireless EMG-device (Myon 320, myon AG, Schwarzenberg, Switzerland) at a sampling frequency of 1 kHz and preamplified. The preamplifier had an input impedance of 2 MΩ and a gain of 1000.

The experimental protocol was performed using the Erigo device (Hocoma AG, Volketswil, Switzerland) which is a tilt table with integrated robotic stepping mechanism and allows simultaneous verticalisation and passive stepping [40]. It is being used for an early mobilization in patients with neurological, orthopedic, or cardiopulmonal disorders and moves the lower limbs in an almost physiological manner regarding kinematic and kinetic parameters [40]. Through an adjustment of the verticalisation level, a potential instability of the cardiopulmonary system in such patients can be dealt with.

The study procedure was based on preliminary test measurements, according to earlier studies [13, 37]: Muscle activation was recorded three times per condition for 30 seconds each (1) on the Erigo, (2) during the stair climbing task itself, and (3) during MI of stair climbing. The stair steps were sufficiently wide enough to allow for heelstrikes. How the subjects performed the footstrikes in order to receive data that derive from heelstrikes or at least mid-footstrikes was visually controlled. The robot-assisted tilt table used in this study provides the options of a progressive verticalisation up to 90° and also guided cyclic leg movements with leg loading. For this study, the movement pattern altering leg with a cadence of 32 steps per minute and a verticalisation to the highest level was used. This verticalisation level provided the best possible approximation to the upright posture during stair climbing. The MI task was conducted in a seated position in order to reduce additional EMG activity that usually occurs due to postural sway during standing with closed eyes [41] and to reduce the risk of falling. For the MI task, a chair was placed in front of the stair. Each actively performed task on the stair was followed by an MI task in the seated position. The stair climbing tasks were performed at a self-selected speed without using the handrails. All participants were asked to imagine stair climbing from an internal perspective and to focus on visual and kinaesthetic accuracy. MI and stair climbing were performed starting with the dominant leg. In all three conditions, a push button connected to the EMG recording system via Bluetooth was used by the participants themselves in order to indicate each footstrike of the dominant leg during stair climbing and the motor imagery task. For the stepping task on the Erigo, participants were asked to signal the moment of the highest foot position or smallest knee flexion angle, roughly comparable with the foot position in the moment of the footstrike during stair climbing.

To ensure a similar level of knowledge on the MI concept, participants received general information prior to the day of data collection. In order to control the MI performance, MI ability was evaluated using three assessments: mental rotation (MR) [42, 43], mental chronometry (MC) [44, 45], and the Kinaesthetic and Visual Imagery Questionnaire [46] in German (short form; KVIQ-10). MR was earlier described as an index of implicit motor imagery where the tested individual is asked to imagine how an object would look rotated from the actual orientation [47]. MC is a valid and reliable assessment to test the temporal congruency between real and imagined movements in healthy and poststroke patients [48].

*Data Analysis*. The recorded EMG data were rectified, high-pass filtered at 10 Hz, and smoothed with a moving average of 100 ms. As a next step, the data were cut according to the push button signals that indicated all single footstrikes. A step cycle thus started and ended with a footstrike. As expected, the step cycles revealed a slightly different duration both within and between the subjects. Therefore, a time-normalization was applied to scale down every data set to an equivalent size of 1000 frames per step cycle using a linear interpolation function. On the basis of these time-normalized data sets the EMG profiles of the mean step cycle for each subject could be calculated separately for each condition and muscle. The processed data were further subjected to a calculation of the cross-correlation coefficient at zero time lag to test for similarity/dissimilarity [49] for each of the four muscles between the conditions. Cross-correlation is an established method and was already frequently used in several studies, for instance, to compare EMG signals from different walking trials and different test sessions of healthy subjects for evaluating the cross-correlation itself in that context [49], to test for lower limb muscle coactivation during walking at different speeds also in healthy subjects [50] or abnormal muscle activation patterns in patients with gait deficits after traumatic brain injury [49]. The cross-correlation coefficient is a measure of the similarity between two curves. It is sensitive to similarities or dissimilarities in temporal characteristics and, when there is no time lag, to similarities and differences in shape [49]. The cross-correlation value (R) can vary between 0 and 1 and tends towards 1 in case a pair of curves is of the same shape. The averaged EMG profiles per muscle, participant, and condition were planned to be used for the cross-correlation analysis. The according coefficient (see (1)) was calculated as follows:

(1) $$\begin{array}{c} R=\frac{\sum x_{i}y_{i}}{{\left(\sum x_{i}^{2}\right)}^{1/2}{\left(\sum y_{i}^{2}\right)}^{1/2}} \end{array}$$

with *x*_*i*_ and *y*_*i*_ as the two series [49].

The cross-correlation compares two series in terms of timing and shape but not amplitude. Thus, additionally and as a secondary parameter the average EMG activation amplitudes of the three conditions were compared first (1). This was followed by a comparison of the average EMG amplitudes of the motor imagery and a resting condition (2) in order to decide whether to include the MI data in the cross-correlation calculation (3) which was the primary outcome parameter.

## 3. Results

**Figure 1:**
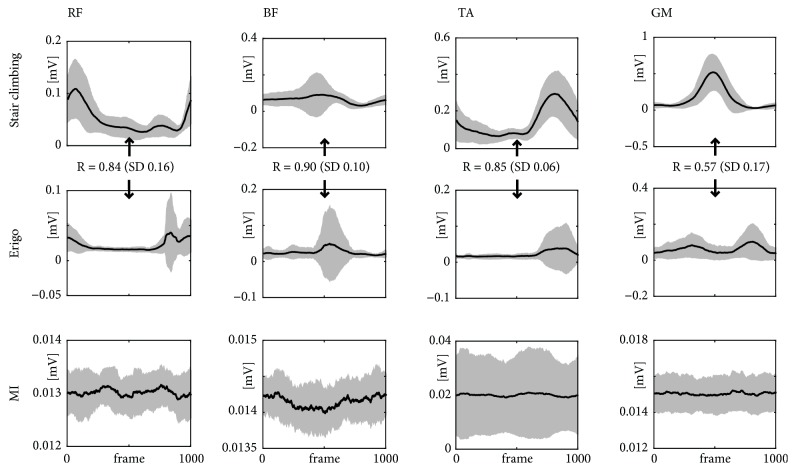
Grand mean and SD of the time-normalized EMG activity. It contains the data of all 12 subjects for the different conditions and the four leg muscles of the dominant side; RF: m. rectus femoris, BF: m. biceps femoris, TA: m. tibialis anterior, and GM: m. gastrocnemius medialis. Cross-correlation coefficients and SD values for the comparison between stair climbing and Erigo EMG profiles as a measure of similarity regarding shape and timing (R = 0 no similarity and R = 1 perfect similarity).

(1) The Shapiro–Wilk test revealed that the data were not normally distributed. Accordingly, the comparison was performed using the Friedman Test followed by Wilcoxon rank sum post hoc analysis with Bonferroni correction. As expected, all four muscles showed a highly different average EMG activation amplitude (see Figure 1) across the three conditions (p < 0.001). The post hoc analysis revealed that the mean amplitudes for all muscles and conditions differed significantly with p < 0.01 (or p < 0.05 for the comparison between stair climbing and Erigo for the data from m. gastrocnemius medialis).

(2) The results of the MI ability assessments are presented in Table 1. They reveal that all participants were well able to perform the MI tasks. The comparison of the average EMG amplitude between MI and the resting phase using the Wilcoxon rank sum test showed that there was no difference in any of the muscles (p > 0.05). Accordingly, the data of the MI condition were not subjected to a further comparative analysis, i.e., the cross-correlation calculation.

(3) On average, the cross-correlation coefficients of the comparison of stair climbing and stepping on the Erigo indicated a high similarity of the EMG activation for three muscles: m. rectus femoris (R=0.84 ± 0.16), m. biceps femoris (R=0.90 ± 0.10), and m. tibialis anterior (R= 0.85 ± 0.06). For the m. gastrocnemius medialis only a moderate similarity (R=0.57 ± 0.17) across the participants could be found.

## 4. Discussion

The restoration of gait ability following stroke is a major task in rehabilitation and the promotion of physical activity has become a significant element in the management of rehabilitation after stroke as survivors often experience physical deconditioning and lead sedentary lifestyles [51]. For more than a decade, robotic devices have been integrated into neurorehabilitation programs with promising results. For instance, initial work on robotically driven gait orthosis has revealed that walking within an automated exoskeleton is not entirely passive [52, 53]. Furthermore, it has been found that poststroke patients who practice bodyweight supported treadmill training show an improved electromyographic activity during locomotion [54, 55]. For the Erigo which is the only tilt table that provides a stepping function the electromyographic pattern has not yet been assessed before with the goal of quantifying the similarity to the pattern that derives from stair climbing. Therefore, the aim was to obtain first hints on the possible advantage of this rehabilitation device as well as of motor imagery as accompanying measures to redevelop the stair climbing ability in patients with neurologically based movement disorders.

The main finding of this study is the presence of similar electromyographic activation patterns with regard to timing and shape of the EMG curves during stepping on the Erigo compared to the actual performance of stair climbing. For the selected leg muscles the degree of similarity was predominantly high with somewhat lower correlation coefficients for one of the muscles (m. gastrocnemius medialis). Our data are in line with a previous study using a different gait robot (G-EO-Systems) (EO, Lat:I walk) which provides partial weight support but not a variable verticalisation function for patients with instability of the cardiopulmonary system as the Erigo. In their study, Hesse and colleagues also found comparable lower limb activation patterns during real and simulated conditions [37].

The importance of our finding should be interpreted within the understanding that we are faced with the challenge of a growing percentage of elderly and impaired patients resulting in the need for diverse rehabilitation measures in order to maintain or regain substantial abilities such as independent stair climbing. In this respect, it is clearly important to also apply a variety of rehabilitation technologies and techniques that might complement the range of available possibilities used in rehabilitation besides the direct personal treatment from therapists. As for the stair climbing ability, both investigated methods were considered promising and worthwhile to be integrated into the study. However, it was nevertheless questionable whether motor imagery of stair climbing would result in a specific EMG pattern, and the absence of any overt EMG activity during MI is indeed in line with previous studies [31, 56–58]. It was proposed before that it might be caused by an inhibitory effect of MI on muscle activation in order to block motor output before reaching the motor neuron level [59]. Motor activation during MI might also be subliminal and therefore insufficient to fire spinal motoneurons [60]. Although no peripheral activation could be recorded during MI, the motor learning process which MI aims at is explained on the other hand due to internal stimulation of movements generating pseudo-proprioceptive information which forms the basis for motor improvement [31]. However, the MI assessments showed that all participants had the capacity to mentally represent the stair climbing movement (Table 1) which in turn did not result in MI related activity. Yet, as there was not any detectable EMG activity in the motor imagery condition it seemed unnecessary to further proceed with these data.

In contrast, the results of the comparison between stair climbing and the activity on the Erigo seem promising. Obviously, there is a quite strong similarity with respect to timing and shape of the according EMG patterns indicating the possible use of the Erigo for the promotion of the relearning process of stair climbing. On the basis of the results, we assume that it would be worthwhile to investigate the instantaneous and the long-term effect of a robot-assisted tilt table practice using the Erigo on the EMG activation pattern in neurologic patients with lower limb impairment. It has already been shown that alterations in the muscle activation patterns can be evoked when using robotic devices for walking [61]. The goal would accordingly be (1) to see how the patterns of patients deviate from those of healthy persons and (2) whether after a relearning process the patterns return to those of healthy controls and insofar to evaluate whether stepping on the Erigo performed by such patients might help to regain a more similar EMG pattern as compared to healthy individuals. Thus, it could give more hints on the specific usage of this rehabilitation method as a preparatory measure that potentially supports and facilitates the relearning process of stair climbing. With regard to a possible follow-up study on patients with an impaired motor function, the intention of the current study was not only to obtain initial measurements data from healthy participants but also to evaluate the feasibility of this measurement setup. A subsequent study could insofar focus on severely affected patients who rely on this specific robot-assisted tilt table as the Erigo is the only device that provides the option of a verticalisation combined with a stepping function.

*Limitations of the Study*. Although the findings provide a first insight into the comparability of the EMG activation of the conditions examined here, it should be taken into account that the study population was not gender-balanced. It is known that gender differences exist in walking affecting various gait parameters [62–67]. For instance, gender-related differences in myoelectric activity, i.e., in the occurrence frequency of sEMG of ankle muscles, have been found [68]. Although the cross-correlation values were calculated within each participant it might be considered a limiting factor when generalizing the study findings. Furthermore, the heelstrikes were not technically detected both in stair climbing and on the Erigo but were indicated by the participants themselves which might have caused an error with respect to the timing of the actual heelstrike. In order to achieve a minimization of the error, the data were checked for outliers in terms of step duration.

### 4.1. Conclusion

It was shown that robot-assisted tilt table stepping on the Erigo can evoke a similar EMG activation in certain leg muscles of healthy adults compared to real stair climbing. This finding might provide further evidence in favour of using this therapeutical measure in order to potentially provoke an activation pattern that roughly corresponds to the pattern of healthy individuals. It also suggests an evaluation of potential neuromuscular changes in patients with lower limb motor impairment after stroke practicing robot-assisted tilt table stepping with regard to a more selective use of this therapy. On the other hand, performing motor imagery of stair climbing did not result in overt changes in the EMG activity. This, in turn, does not necessarily mean that motor imagery of this specific movement is ineffective as it might be effective in other levels of the motor system.

## Figures and Tables

**Table 1:** Characteristics of the participants. G (gender): m: male; f: female; PA (physical activity level): 1: rarely/minimal, 2: often/moderate, and 3: frequent/intense; MI exp. (MI experience): y: yes; n: no; Hand (handedness): r: right; l: left; MR (mental rotation): number of correct identified stimuli, total=3; SP (choice of spontaneous perspective): i: internal; e: external; KVIQ v (KVIQ-10 visual domain): total=25; KVIQ k (KVIQ-10 kinaesthetic domain): total=25; KVIQ total (KVIQ-10 total score): total=50; MC PE (mental chronometry, physical execution): time in seconds; MC MI (mental chronometry, motor imagery): time in seconds; MI:PE: ratio of motor imagery and physical execution; vis mean, kin mean: average value of self-rating after each trial during MI (visual and kinaesthetic subscale 1-5); ^*∗*^mean ± SD.

ID	Age	G	PA	MI	Hand	MR	SP	KVIQ	KVIQ	KVIQ	MC	MC	MI:PE	vis	kin
				exp.				v	k	total	PE (s)	MI (s)		mean	mean
1	37	m	2	n	r	32.0	i/i	18	18	36	4.9	5.1	1.0	3.7	3.3
2	58	f	1	n	r	29.0	i/i	19	14	33	4.6	5.4	0.9	3.7	3.7
3	30	f	1	n	r	31.0	i/i	18	2	38	6.1	5.9	1.0	4.0	4.3
4	33	f	3	y	r	32.0	i/i	21	18	39	7.3	7.9	0.9	3.0	4.0
5	38	m	2	n	r	29.0	e/i	13	19	32	6.7	6.5	1.0	2.7	2.7
6	41	f	2	y	r	32.0	i/i	19	19	38	7.9	9.2	0.9	2.7	2.7
7	53	f	2	n	r	29.0	i/i	14	17	31	5.3	6.5	0.8	3.3	3.3
8	67	m	3	n	r	32.0	i/i	11	17	28	5.6	5.4	1.0	1.7	3.7
9	59	f	3	y	r	27.0	i/i	12	19	31	5.4	6.1	0.9	3.7	5.0
10	48	f	2	y	r	31.0	i/i	22	25	47	7.0	9.2	0.8	3.3	4.7
11	67	f	2	n	l	26.0	i/i	19	18	37	5.5	7.1	0.8	3.7	3.0
12	73	m	2	n	r	28.0	i/i	15	15	30	4.9	5.1	1.0	4.0	4.0

Total	50.3	f:		y: n=4	r:			16.8	18.3	35	5.9	6.6	0.9	3.3	3.7
	±14.6	n=8		n: n=8	n=11			±3.6	±2.7	±5.23	±1.1	±1.5	±0.1	±0.7	±0.7
		m:			l:										
	*∗*	n=4			n=1			*∗*	*∗*	*∗*	*∗*	*∗*	*∗*	*∗*	*∗*

## Data Availability

All EMG raw data are available from the Open Science Foundation database (https://osf.io/dwgc7/).
